# Emergent long-range synchronization of oscillating ecological populations without external forcing described by Ising universality

**DOI:** 10.1038/ncomms7664

**Published:** 2015-04-08

**Authors:** Andrew E. Noble, Jonathan Machta, Alan Hastings

**Affiliations:** 1 Department of Environmental Science and Policy, University of California, Davis, California 95616, USA; 2 Department of Physics, University of Massachusetts, Amherst, Massachusetts 01003, USA; 3 Santa Fe Institute, 1399 Hyde Park Road, Santa Fe, New Mexico 87501, USA

## Abstract

Understanding the synchronization of oscillations across space is fundamentally important to many scientific disciplines. In ecology, long-range synchronization of oscillations in spatial populations may elevate extinction risk and signal an impending catastrophe. The prevailing assumption is that synchronization on distances longer than the dispersal scale can only be due to environmental correlation (the Moran effect). In contrast, we show how long-range synchronization can emerge over distances much longer than the length scales of either dispersal or environmental correlation. In particular, we demonstrate that the transition from incoherence to long-range synchronization of two-cycle oscillations in noisy spatial population models is described by the Ising universality class of statistical physics. This result shows, in contrast to all previous work, how the Ising critical transition can emerge directly from the dynamics of ecological populations.

## Abstract (web summary)

Long-range synchronization of ecological populations separated by distances greater than their dispersal range is thought only to occur via environmental correlations. Here, Noble *et al.* show that synchronization can also occur beyond these distances, and is described by the Ising universality class.

## Graphical abstract

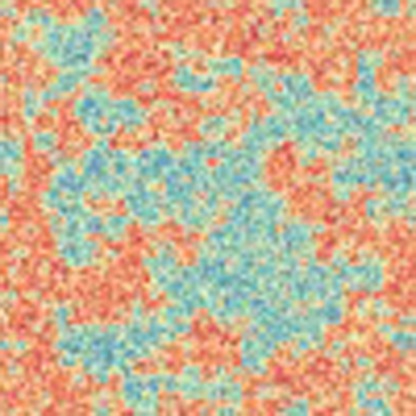

Synchrony of dynamics across space has been a subject of intense study across a diverse array of scientific disciplines^1^,^2^,^3^,^4^,^5^. The study of synchrony of oscillations in population numbers across space and time has a long history in ecology^6^ and has provided great insights into fundamental issues of population dynamics^3^,^5^. The absence of spatial synchrony is generally considered to be the key to persistence in exploiter–victim systems^5^,^7^,^8^,^9^, and may be vitally important in the conservation of species^3^,^5^,^7^ and disease eradication^10^. There is long-standing recognition of the need for new models and measures of spatial synchrony relevant to ecology^3^,^5^,^10^,^11^ that do not depend upon the simplifying assumptions of the Kuramoto^1^,^12^ and related models^2^,^4^.

Three well-known causes of spatial synchrony are the long-range correlation of environmental variation (known as the Moran effect^6^), entrainment to neighbouring populations via trophic interactions and dispersal of individuals among habitat patches^3^,^5^,^13^. In the absence of environmental noise, short-range dispersal is sufficient to phase-lock spatial populations over arbitrarily large distance scales^7^. In the more realistic case of environmental variation, short-range dispersal has been shown to synchronize population oscillations only over distance scales comparable to the correlation length of the environmental noise^5^,^14^,^15^,^16^. The remaining question is whether and how synchrony over distances much longer than the scale of environmental correlations can arise from short-range dispersal in the presence of strong noise. This paper is the first to demonstrate the emergence of long-range synchronization and model-independent power-law scalings in noisy cyclic populations. Note that our results apply to the onset of spatial synchrony of oscillations in population numbers far from the extinction threshold, and our work is distinct from previous investigations of model independent power laws near the extinction threshold^17^,^18^,^19^.

Many of the most prominent studies of spatial synchrony have focused on examples where the underlying dynamics are two cycles, including the dynamics of childhood diseases^10^ and small mammals^13^. Because both of these examples have been argued to arise in part from the seasonal forces that impact the vast majority of ecological populations, two-cycle behaviour is truly a ubiquitous phenomenon. That two cycles are so common is expected from the structure of overcompensatory models in population biology, such as the logistic map, because of the large range of growth rates for which the asymptotic dynamics lie in an exact two-cycle. With the addition of noise, approximate two-cycle behaviour can be found over an even larger range of growth rates^20^ along the entire period-doubling sequence and through the two-banded chaotic region, as can be seen in the bifurcation diagram for any quadratic map^21^.

In statistical physics, the canonical model for the emergence of long-range order from the collective behaviour of local interactions is the Ising model^22^,^23^,^24^,^25^. The Ising model describes emergent phenomena at a phase transition where a simple twofold symmetry is broken. Physical systems with unrelated microscopic dynamics, such as magnetic, liquid–vapour and binary alloy systems, exhibit the power-law scaling behaviour of the Ising model at a critical phase transition. The Ising model thus defines a ‘universality class'—a set of many disparate systems that are all characterized by identical power-law scaling behaviour over large distances despite wide differences in local interactions and short-range behaviour. Here, for a suite of ecological models, we will show that the power-laws describing asymptotic behaviour at the emergence of long-range synchronization are in the two-dimensional (2D) Ising universality class, providing a powerful tool for understanding the emergence of collective synchronization in spatial populations with local dispersal.

This quantitative correspondence between ecological population models and the Ising model is quite surprising and novel. The Ising universality class describes phase transitions for systems in thermal equilibrium, that is, systems with a well-defined energy and temperature^22^,^23^,^24^,^25^. On the other hand, ecological population models are dynamical, dissipative systems lying far from thermal equilibrium. Previous attempts to apply the Ising model to population ecology simply assume thermal equilibrium and lack any clear ecological motivation. In this paper, we show how a quantitative correspondence to the Ising model emerges naturally in spatially extended dynamical models of ecological populations, providing new insights for both fields.

Here, we first introduce a suite of discrete-time spatial population models and define a statistic for discriminating between incoherent and synchronous behaviour. Next, we demonstrate that the asymptotic behaviour of ecological population dynamics at the emergence of long-range synchronization is described by the Ising universality class. Finally, we define new statistics of spatial synchrony, each corresponding to a statistic of the Ising model, that can rapidly detect sudden changes in the spatial synchrony of ecological populations. We anticipate that our methods will be broadly applicable to the study of transitions in the spatial synchrony of cyclic populations, and we underscore the importance of universality for ecological modelling.

## Results

### Definition of the synchronization order parameter

We investigate the long-range synchronization of two-cycle oscillations in several discrete-time spatial population models with local dispersal and uncorrelated environmental noise: a single-species Ricker model (Methods equations (4) and (5))^26^,^27^ with two different dispersal kernels, a single-species Logistic model (Methods equations (4) and (6))^28^,^29^,^30^,^31^ and a version of the Nicholson–Bailey Host–Parasitoid model (Methods equations (7) and (8))^32^. The analogue of the local ‘spin' variable at a lattice site in the Ising model is a local two-cycle amplitude, *m*_*j*,*t*_ (Methods equation (9)), defined at each habitat patch *j* and generation *t* in the spatial population models. A spatiotemporal average of the *m*_*j*,*t*_ is the synchronization order parameter, *m* (Methods equation (11)), which discriminates between phases of incoherence and long-range synchronization: an order parameter value of zero signals complete incoherence (the disordered phase), while nonzero values measure the degree of long-range synchronization (the ordered phase). On a landscape with one or two spatial dimensions (one-dimensional (1D) or 2D) and *L* habitat patches along each side (such that the total number of habitat patches is *N*=*L* or *N*=*L*^2^, respectively), finite-size estimates of the synchronization order parameter, *m*_*L*_ (Methods equation (13)), can be obtained from numerical simulations.

### Ising universality at the critical transition

**Figure 1:**
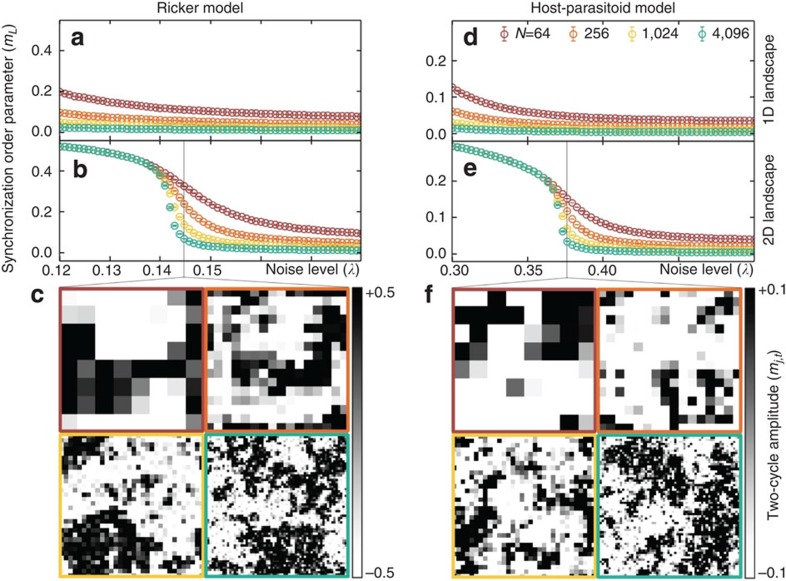
Critical transitions at the onset of collective synchronization in spatial Ricker and host–parasitoid models on 2D landscapes. (**a**,**d**) On large 1D landscapes (large *N*=*L*) with short-range dispersal and nonzero uncorrelated environmental noise levels (*λ*), collective synchronization of spatial populations cannot emerge from local dispersal alone. (**b**,**e**) On 2D landscapes (where *N*=*L*^2^), collective synchronization can emerge from local dispersal at a noise-induced transition. A continuous change in numerical estimates of the synchronization order parameter, *m*_*L*_, from near zero (the disordered, incoherent phase) to larger values (the ordered, synchronous phase), sharpens as the size of the landscape increases. (**c**,**f**) Near the transition, on 2D landscapes ranging in size from *N*=64 (upper-left) to 4,096 (lower-right), density plots of local two-cycle amplitudes, the *m*_*j*,*t*_, show emergent long-range order in the fractal coexistence of synchronized habitat patches. In statistical physics, a continuous transition from a disordered phase to a phase ordered by emergent long-range correlations defines a ‘critical transition'. Critical transitions at the onset of collective synchronization also occur in the 2D Logistic and Ricker–Moore models. Note that symbols are displayed with s.e.m. error bars, but actual error bars are often much smaller than the symbol size.

We present results for 1D and 2D ecological models with various numbers of habitat patches. The synchronization order parameter as a function of uncorrelated environmental noise level is plotted in Fig. 1a,b,d,e and Supplementary Figs 1a,b and 2a,b. On 1D landscapes, for sufficiently large *N*, the order parameter approaches zero in all the population models, and we find no evidence of long-range synchronization at nonzero noise levels. The same behaviour occurs in the 1D Ising model^22^,^23^,^24^,^25^. The behaviour on 2D landscapes is quite different. A continuous transition in the synchronization order parameter sharpens with increasing landscape size. Near this ‘critical point', synchronized habitat clusters appear on the landscape and form fluctuating fractal patterns (Fig. 1c,f; Supplementary Figs 1c and 2c) associated with the emergence of long-range order and critical slowing down (Supplementary Fig. 3). These results demonstrate both that long-range synchronization can be an emergent phenomenon on 2D landscapes even in the presence of noise, and also that the existence of a critical transition from incoherence to synchrony is independent of the details of the local ecological dynamics.

**Figure 2:**
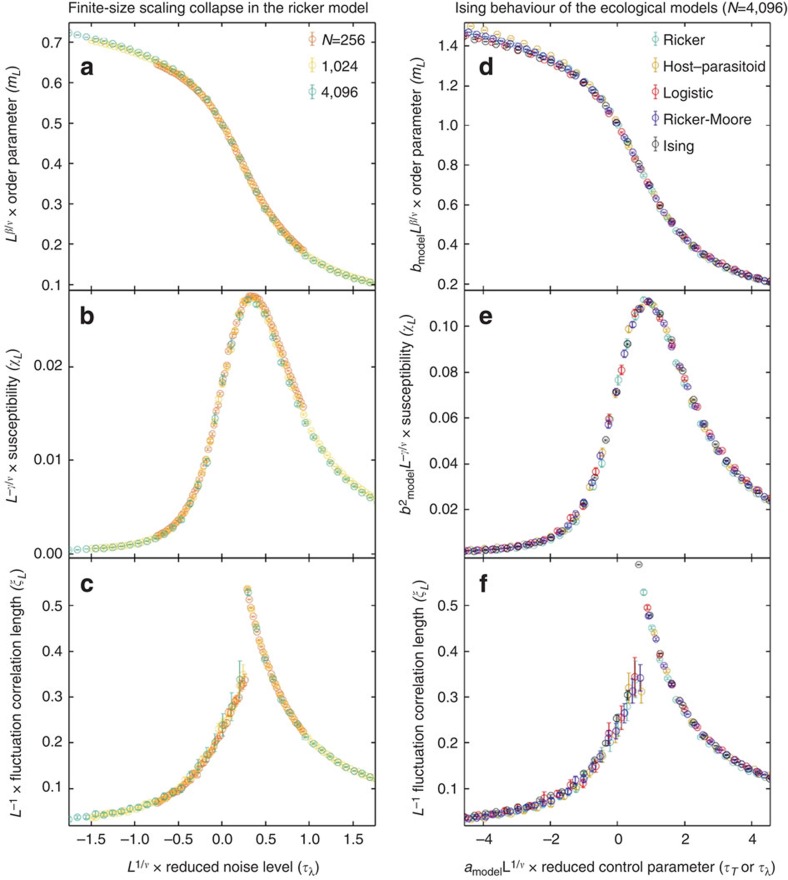
Ising universality near the onset of collective synchronization in ecological populations on 2D landscapes. Scaled measurements of the order parameter (*m*_*L*_), susceptibility (*χ*_*L*_) and fluctuation correlation length (*ξ*_*L*_) for diverse ecological models and 2D landscape sizes (where *N*=*L*^2^) coincide with Ising model results, showing the existence of universal behaviour. (**a**–**c**) Finite-size scaling collapse (as a function of *L* and the Ising critical exponents, *ν*=1, *β*=1/8, *γ*=7/4) demonstrates universal Ising behaviour of the Ricker model over a range of ecologically relevant landscape sizes. The same finite-size scaling behaviour is found in all the ecological models that we analyse. (**d**–**f**) Scaled collapse of ecological statistics onto the corresponding Ising statistics for *N*=4,096. Scaling parameters, *a*_model_ and *b*_model_, are chosen separately for each model; without loss of generality, *a*_Ising_=*b*_Ising_=1. These results present strong evidence that the onset of collective synchronization is a critical transition in the Ising universality class. Universality explains the robustness and broad applicability of our results: long-range synchronization and power-law scalings emerge from local dispersal independent of the details of local dynamics, including population regulation, dispersal and landscape connectivity. Note that symbols are displayed with s.e.m. error bars, but actual error bars are often much smaller than the symbol size.

We now quantify the strong correspondence between the critical behaviour of the Ising model and the emergence of long-range synchronization on 2D landscapes. In addition to finite-size estimates of the order parameter, *m*_*L*_ (Methods equation (13)), we define two statistics of the local two-cycle amplitudes motivated by the Ising correspondence: the *susceptibility*, *χ*_*L*_ (Methods equation (14)), measures the variance of two-cycle amplitudes, and the fluctuation correlation length, *ξ*_*L*_ (Methods equation (16)), measures the length scale of long-range spatial correlations among two-cycle amplitudes. We find ‘finite-size scaling collapses' in which measurements of *m*_*L*_, *χ*_*L*_ and *ξ*_*L*_ on landscapes of different sizes and noise levels, when multiplied by appropriate powers of *L* given by the Ising ‘critical exponents', lie on universal curves (Methods equation (24); Fig. 2a–c; Supplementary Figs 4–7). We also identify a scaling collapse across models: statistics of the different ecological population models, multiplied by model-dependent pre-factors, collapse onto corresponding Ising statistics near the critical transition (Methods equation (24); Fig. 2d–f; Supplementary Figs 8 and 9).

While it is not the main focus of our work, the slow dynamics near the Ising critical point is also ecologically interesting. The Ising model equipped with the relevant non-conservative dynamics is referred to as ‘model A'^33^,^34^,^35^. Critical dynamics described by model A are universal and known to hold across a wide spectrum of physical systems near and far from thermal equilibrium^36^,^37^,^38^,^39^,^40^,^41^,^42^,^43^,^44^,^45^,^46^. We expect that the critical dynamics of all of the spatial population models studied here fall into the model-A dynamic universality class.

Ising universality explains the robustness and broad applicability of our results and provides a quantitative description of scaling behaviour at the onset of long-range synchronization that is independent of the details of local population dynamics. In particular, universality allows us to conclude that long-range synchronization among ecological oscillators can emerge from short-range dispersal even when a detailed description of the local dispersal behaviour and dynamics is unknown. We have explicitly demonstrated identical scaling behaviour in the spatial Ricker model with either the nearest-neighbour or Moore neighbourhood dispersal kernel (Fig. 2; Supplementary Figs 4,7 and 9), and universality ensures that the same scaling behaviour would be found for any localized dispersal kernel. Ecological populations exhibit even greater variability in their local connectivity but are nonetheless expected to be in the (random) Ising universality class^23^ as long as dispersal remains short-ranged.

**Figure 3:**
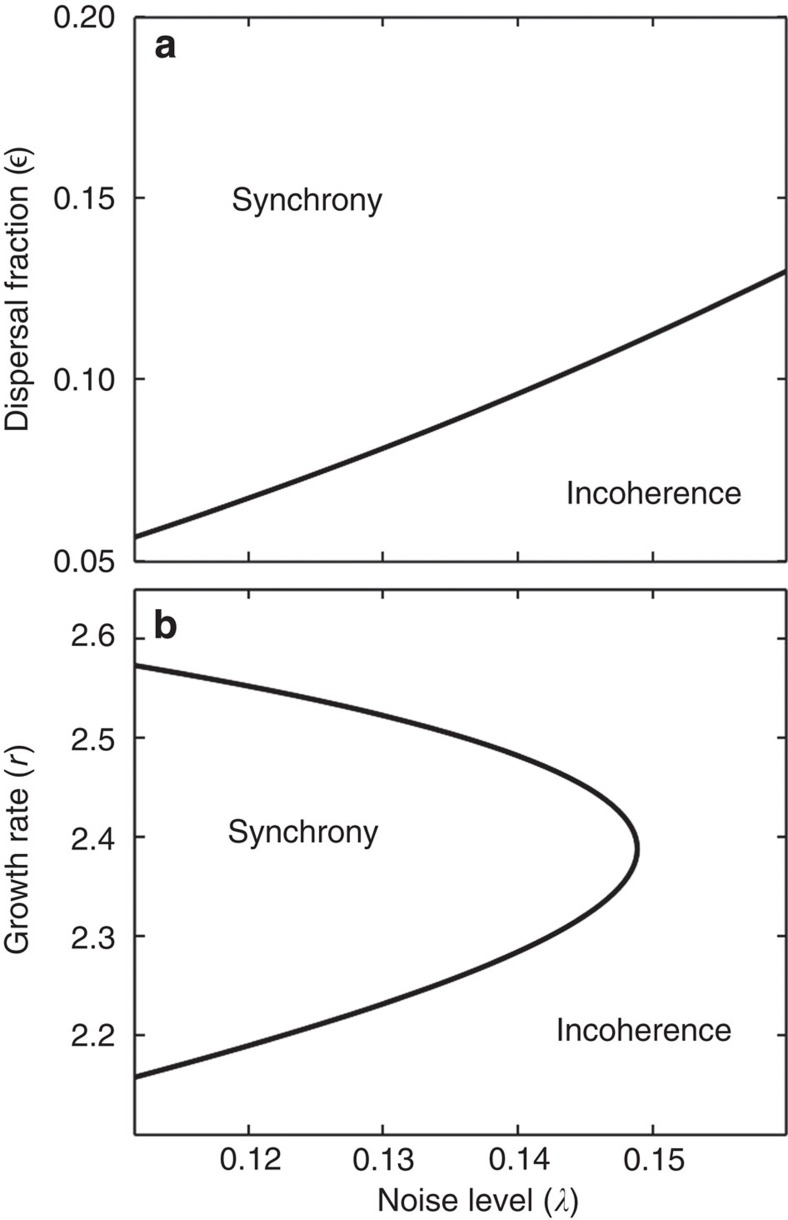
Phase diagram for incoherence and collective synchronization in the Ricker model on 2D landscapes. The parameters of the Ricker model are dispersal fraction 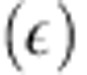, growth rate (*r*) and noise level (*λ*). For any given choice of parameters, there is a single asymptotic phase: either incoherence (with synchronization order parameter zero) or synchrony (with synchronization order parameter nonzero). These two phases are separated by a boundary of Ising critical transitions. For (**a**) fixed growth rate and (**b**) fixed dispersal fraction, we plot cross-sections of the critical boundary. Note that long-range synchronization can emerge from local dispersal despite strong uncorrelated environmental noise. A ‘reentrant transition' from synchrony to incoherence at high growth rate, as seen in **b**, is associated with the disapperance of two-banded behaviour in the bifurcation diagram of the underlying Ricker map.

We can determine the conditions for long-range synchronization as a function of the parameters in each ecological model. For the spatial Ricker model, this information is summarized in Fig. 3 as a ‘phase diagram' for incoherence and synchrony (see also Supplementary Fig. 10). We find that long-range synchronization emerging from local dispersal can persist in the presence of strong uncorrelated environmental noise. We conjecture that all critical points on the phase boundary lie in the 2D Ising universality class. The shape of the phase boundary as a function of growth rate, dispersal fraction and noise level will be qualitatively similar in the other population models.

### Rapid detection of sudden changes in spatial synchrony

Given that large-scale disturbances are not uncommon, the study of transient dynamics is especially important in ecology^47^. The rapid formation of complex patterns of spatial synchrony can be difficult to infer on timescales short enough to allow for an effective response. Using the relatively short time-series data typically available, new measures are needed to rapidly detect sudden changes in spatial synchrony. We introduce three such measures, each corresponding to a statistic of the Ising model: the synchronization index, *S*_*t*_ (Methods equation (17)), measures instantaneous synchronization as a non-spatial, two-generation, simple moving average; the susceptibility index, *V*_*t*_ (Methods equation (19)), measures the magnitude of fluctuations in synchronization windowed in both space and time; the correlation length index, CL_*t*_ (Methods equation (23)), measures a distance scale proportional to the size of synchronized clusters and is also windowed in both space and time. The spatial windowing of *V*_*t*_ and CL_*t*_ allows statistical power to be gathered across space in the absence of a long time series^48^.

**Figure 4:**
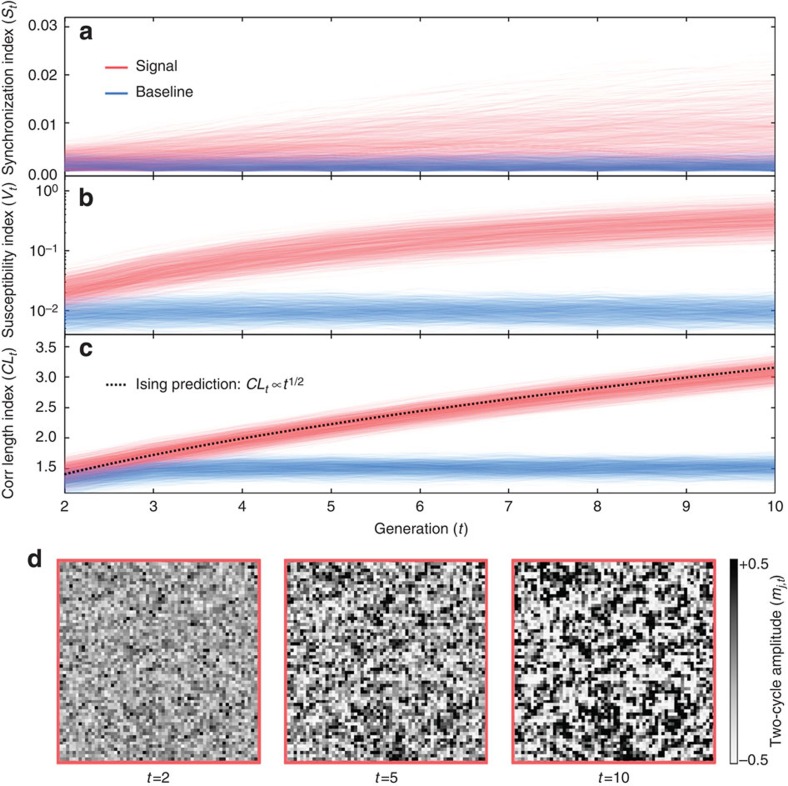
Rapid detection of sudden changes in spatial synchrony from incoherence to synchronized cluster formation and growth. Synchronized cluster formation and growth in ecological models is analogous to a ‘quench' in the dynamical Ising model. Starting with an ensemble of 1,000 incoherent population configurations at generation zero (*t*=0), we calculate trajectories of the (**a**) synchronization index (*S*_*t*_), (**b**) susceptibility index (*V*_*t*_) and (**c**) correlation length index (CL_*t*_) emerging from the dynamics of a Ricker model on a 2D landscape of *N*=*L*^2^=4,096 habitat patches. The growth rate, dispersal fraction and noise level correspond to either the synchronous phase (the signal, red trajectories) or the incoherent phase (the baseline, blue trajectories). Each index is averaged over two generations and can be calculated starting at *t*=2. The spatially windowed indices, *V*_*t*_ and CL_*t*_, outperform the non-spatial index, *S*_*t*_, and allow for rapid detection of a sudden change in spatial synchrony within two or three generations. (**d**) Snapshots at *t*=2, 5 and 10 of local two-cycle amplitudes, the *m*_*j*,*t*_, are shown along a representative trajectory of the Ricker model with parameters corresponding to the synchronous phase. Sharp ecological boundaries between clusters of habitat patches with opposite-sign synchronization can emerge spontaneously on 2D landscapes with local dispersal and uncorrelated environmental noise.

We demonstrate the application of these new measures to the rapid detection of sudden changes in spatial synchrony from incoherence to synchronized cluster formation and growth. In Fig. 4, starting with an ensemble of incoherent population configurations at generation zero (*t*=0), we calculate trajectories of *S*_*t*_, *V*_*t*_ and CL_*t*_ emerging from the dynamics of a Ricker model where the growth rate, dispersal fraction and noise level correspond to either the synchronous phase (the signal, red trajectories) or the incoherent phase (the baseline, blue trajectories). The non-spatial index, *S*_*t*_ (Fig. 4a), is outperformed by the spatially windowed indices, *V*_*t*_ and CL_*t*_ (Fig. 4b,c), each of which allows for detection of a sudden change in spatial synchrony within two or three generations.

Synchronized cluster formation and growth in ecological models is analogous to a ‘quench' in the Ising model with ‘non-conserved' dynamics, where the size of clusters increases according to a power-law in time^22^,^23^,^24^,^25^. This power-law is universal^22^,^23^,^24^,^25^, and therefore, independent of the details of local dynamics. The same power-law behaviour can be found in the Ricker model (Fig. 4c) and in the host–parasitoid model of Rost *et al.*^32^ These observations suggest that the sudden, spontaneous emergence of sharp ecological boundaries between clusters of habitat patches with opposite-sign synchronization, as seen in Fig. 4d, might occur in many types of ecological systems with local dispersal and uncorrelated environmental noise.

## Discussion

By demonstrating the existence of Ising critical transitions in the spatial synchrony of noisy spatial populations, our work shows, for the first time, how long-range synchronization in ecological systems can emerge in the absence of external forcing (the Moran effect^6^). Beyond the important case of transitions between incoherence and two-cycle synchrony, our results suggest a general correspondence between emergent long-range synchronization in ecology and the universality classes of critical transitions in statistical physics. The methods developed here can be readily generalized and applied to establish those correspondences. This high degree of model independence implies that the possibility of emergent long-range synchronization must be carefully considered when seeking to understand the causes and consequences of spatial synchrony^3^,^5^,^6^,^7^,^8^,^9^,^10^,^11^,^12^,^13^,^14^,^15^,^16^,^27^,^32^,^49^,^50^,^51^,^52^,^53^,^54^,^55^,^56^, including in the dynamics of childhood diseases, such as measles and pertussis^10^, in the biological control of outbreaks of insects, such as bark beetles and gypsy moths^5^ and in the mitigation of synchronous masting, which threatens food security in agro-ecosystems^56^.

Finding appropriate measures of spatial synchrony in 2D ecological systems has been a serious challenge^5^. We have shown how the Ising correspondence introduces new statistics, both asymptotic measures and transient indices, to the study of spatial synchrony. In contrast to the Kuramoto model^1^ and other spatially implicit approaches to quantifying spatial synchrony, these new statistics of spatial synchrony depend on the full spatiotemporal dynamics of a noisy population. Ising-like transient indices should be especially useful in quantifying spatial synchrony over timescales relevant to ecology and in the rapid detection of sudden transitions^57^.

This work and others underscore the vital importance of universality for making robust, quantitative predictions about transitions to long-range order in ecological populations where the full complexity of local dynamics could never be modelled explicitly. Connections between spatial population models and the universality classes of statistical physics have previously been demonstrated in phase transitions to flocking^58^, to extinction^17^,^19^ and to other ordered phases relevant to ecological systems^25^,^59^. Universality of transitions in the spatial synchrony of noisy populations promises to remain a fruitful area of investigation.

## Methods

### Ising model and Ising universality

In ecology, the modelling of spatial populations begins with a dynamical systems approach, while in statistical physics, the modelling of a spin system in thermal equilibrium begins with a probability distribution called the Gibbs distribution^22^,^23^,^24^,^25^


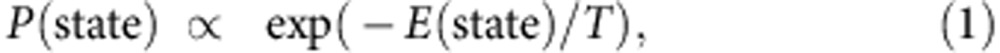


where the spin system is in thermal contact with a thermal reservoir that maintains a fixed temperature, *T*, and the energy of the state is *E*(state). (Boltzmann's constant is set to one in equation (1).) The energy function typically models local interactions among nearest-neighbour spins. In the Ising model^22^,^23^,^24^,^25^, lattice sites are indexed by an integer, *i*, and the local spin, the *s*_*i*_, is either up (+1) or down (−1). The Ising energy function can be written as a sum over products of neighbouring spins


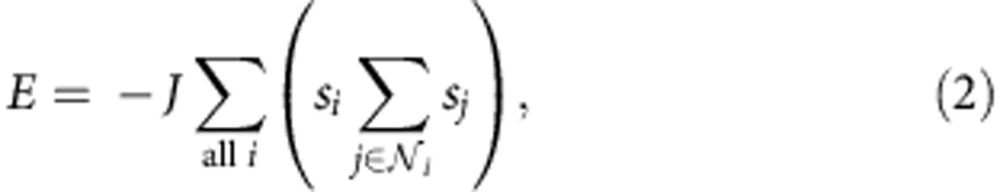


where 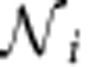 is the set of indices for the neighbouring spins of lattice site *i* and *J* is a coupling constant. The overall negative sign means that the energy is lower (higher) when neighbouring spins are more (less) aligned. Because the probability of any given configuration of local spin states over the lattice is given by the Gibbs distribution, configurations with a high level of local alignment are the most probable at low temperatures. The expected sum of all the spins


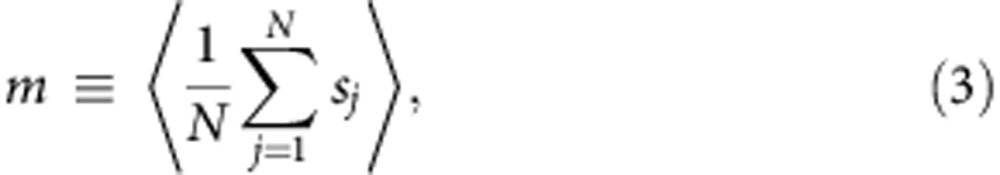


defines the order parameter, *m*. Nonzero values signal an ordered phase in which spins are globally aligned; a zero value signals a disordered phase.

A 1D chain of spins will only order at zero temperature^22^,^23^,^24^,^25^. For 2D lattices, a phase transition from disorder to order occurs at a nonzero critical temperature, where the order parameter goes continuously to zero. Near the critical point, the fluctuation correlation length diverges: clusters of aligned spins form on all length scales, and clusters of opposite-sign spins coexist in a fluctuating fractal pattern. These emergent, long-range correlations render many details of the local interactions unimportant to emergent behaviour near the critical point. Indeed, the critical point of the Ising model is universal: the same power-law scaling relationships are common to critical points observed in seemingly unrelated magnetic, liquid–gas and binary alloy systems^22^,^23^,^24^,^25^, as well as systems far from thermal equilibrium^36^,^37^,^38^,^39^,^40^,^41^,^42^,^43^,^44^,^45^,^46^. The numerical values of the exponents in those power-laws quantitatively define the Ising universality class^22^,^23^,^24^,^25^.

### Ecological population models

We start by investigating long-range synchronization in three models of single-species populations with local dispersal and uncorrelated environmental noise. The population density of habitat patch *j* at generation *t* is a continuous random variable, *X*_*j*,*t*_. The geometry of *N* habitat patches on a spatial landscape is given by a linear or square lattice with periodic boundary conditions and one or two spatial dimensions. (If the linear dimension is *L*, then the landscape size is *N*=*L*^*d*^ where *d*=1, 2.) The spatial dynamics are given by noisy coupled maps


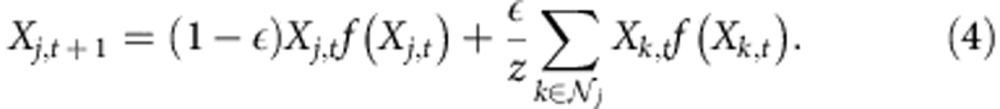


In each generation, local populations are regulated by the per capita density dependence of a noisy quadratic map, *f*(*X*_*j*,*t*_), in which random environmental variation in population regulation, uncorrelated in both space and time, is modelled as multiplicative white noise. Afterwards, a fraction of each local population, 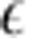, disperses evenly among *z* neighbouring habitat patches, as indexed by the elements of 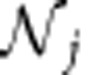, the set of indices for the neighbouring spins of lattice site *j*. Note that changing the number of spatial dimensions does not change the fraction of a local population that disperses.

For any finite-sized landscape and nonzero noise levels, the asymptotic (*t*→∞) phase of the system is extinction (*X*_*j*_=0 for all *j*). Nonetheless, typical extinction times rapidly increase with the number of habitat patches. In numerical simulations for finite *N*, we gather statistics on the single, long-lived, metastable state that is conjectured to be fully stable in the *N*→∞ (or ‘thermodynamic') limit. In the following, ‘asymptotic' will refer to times much larger than one generation but much less than the extinction time.

The Ricker model is equation (4) with a nearest-neighbour dispersal kernel on a linear or square lattice of habitat patches (*z*=2*d*) and local populations regulated by a noisy Ricker map^20^,^26^


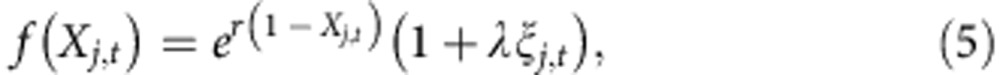


where *r* is the growth rate, *ξ*_*j*,*t*_ are standard normal deviates, uncorrelated in both space and time, and *λ* is the noise level, that is, the s.d. of the environmental noise distribution. Equation (5) is a well-known extension of the per capita density dependence of the Ricker map^26^ to include the effects of multiplicative uncorrelated environmental noise^20^. A similar coupled map has previously been used to understand the causes of intraspecific spatial synchrony in large-scale moth and aphid populations^27^.

The Logistic model is equation (4) with a nearest-neighbour dispersal kernel on a linear or square lattice of habitat patches (*z*=2*d*) and local populations regulated by a noisy logistic map^30^


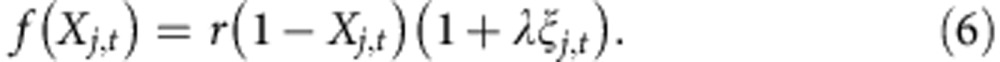


The study of noisy logistic maps has a long history^30^. Pattern formation in coupled logistic maps with local two-cycle oscillations has been investigated previously^31^.

The Ricker–Moore model is the same as the Ricker model but with a broader dispersal kernel. On 1D landscapes, the dispersal fraction is evenly distributed among the nearest and next-to-nearest neighbours (*z*=4). On 2D landscapes, the dispersal fraction is evenly distributed among the habitat patches of the Moore neighbourhood (the *z*=8 nearest and next-to-nearest neighbours on a square lattice).

The host–parasitoid model is a mechanistic two-species model in which the population dynamics of two non-interbreeding cohorts of hosts are coupled together by a shared parasitoid. The spatial dynamics of the host (*H*) and parasitoid (*P*) is taken from equation (8) of Rost *et al.*^32^


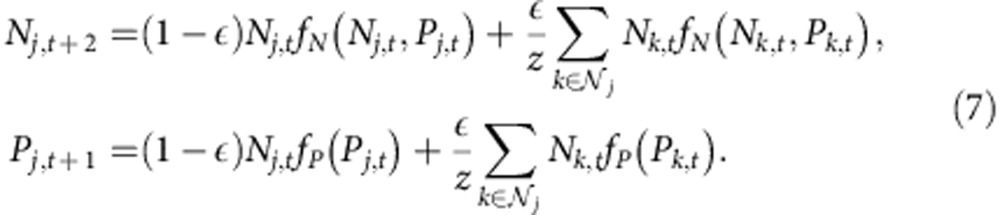


with local populations regulated by a noisy Nicholson–Bailey coupled map


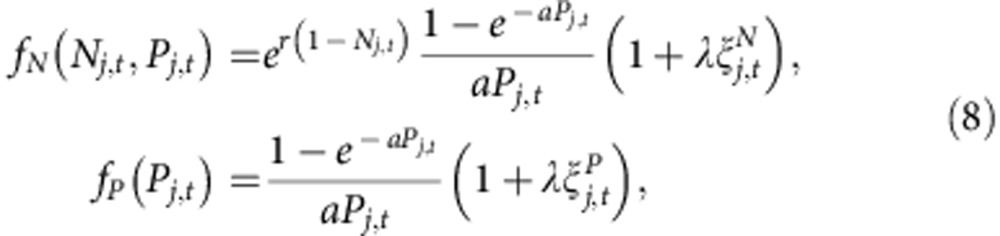


where *r* is the growth rate of the host, *a* is the searching efficiency of the parasitoid, the 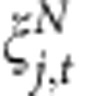 and the 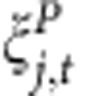 are two sets of standard normal deviates, uncorrelated in space and time and with each other, and *λ* is the noise level. A nearest-neighbour dispersal kernel is assumed, where 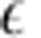 is the dispersal fraction for both host and parasitoid. Rost *et al.*^32^ demonstrated the existence of coarsening behaviour and an asymptotic phase of spatial synchrony. Phase separation and coarsening in two-variable, discrete-time systems with local interactions have also been studied in the physics literature^60^,^61^. Our work focuses on the emergence of long-range synchronization from incoherence at a critical transition and the Ising universality of that transition.

### Synchronization order parameter

In the Ricker, Logistic and Ricker–Moore models, we define a two-cycle amplitude for each local population given by


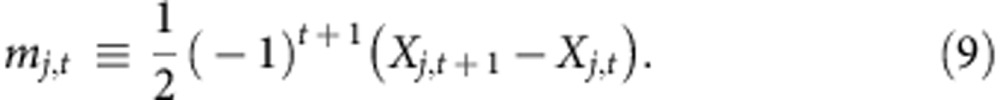


The spatial average of the *m*_*j*,*t*_ defines the instantaneous synchronization


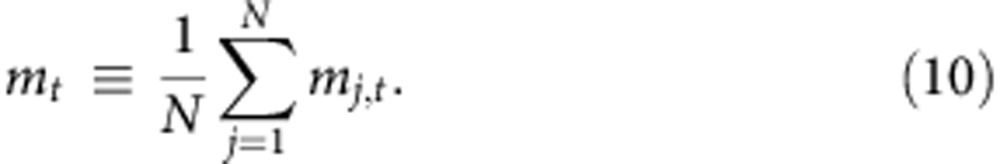


In the physics literature, order parameters are defined by an ensemble average denoted by angle brackets. Here, the expectation value of *m*_*t*_ taken over the probability distribution of the *X*_*j*,*t*_ at long times defines the synchronization order parameter, *m*


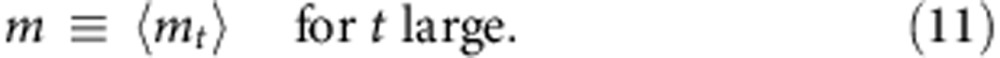


An order parameter value of zero signals complete incoherence (the disordered phase); nonzero values measure the degree of partial synchronization (the ordered phase). The synchronization order parameter of the host–parasitoid model is the same as in equations (9)–(11), 16, 17 but with *N*_*j*,*t*_ substituted for *X*_*j*,*t*_ in the definition of the two-cycle amplitude. In our numerical estimates, we obtain the same results if *P*_*j*,*t*_ is substituted instead. The onset of long-range synchrony in both the host and parasitoid populations is conjectured to occur at the same critical point.

To gather some intuition for the emergence of an Ising critical transition in the synchronization order parameter, we first consider a population in which density, *x*_*t*_, is regulated by the deterministic Ricker map. This is a limiting case of the Ricker model. The limiting value of the two-cycle amplitude, as defined in equation (9), is


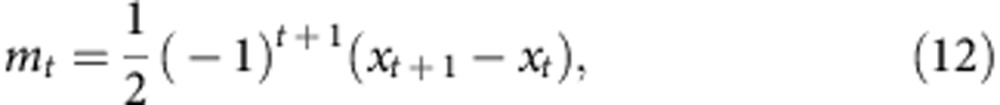


and the limiting value of the synchronization order parameter, as defined in equation (11), is the average value of *m*_*t*_ in the asymptotic regime. For growth rates in the steady-state range, the asymptotic population density is a constant, *x*_*t*_=x*, and the order parameter vanishes, *m*=0. The asymptotic system is clearly invariant under time translations, that is, *t*→*t*+*n* for any integer *n*. For growth rates in the two-cycle range, the asymptotic population density oscillates between two distinct values, 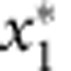 and 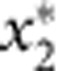. The order parameter takes on one of two possible nonzero values, 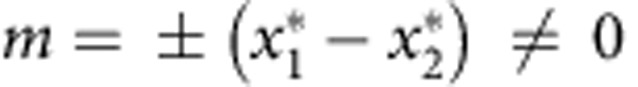, depending on the temporal phase of the two-cycle oscillation. The asymptotic system remains invariant to even-period, but not odd-period, time translations. Therefore, in the deterministic limit, increasing the growth rate from the steady state to the two-cycle range breaks a twofold, or 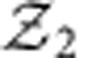, symmetry of the asymptotic steady-state system. In the full Ricker model, high noise levels wash out any cycling in the underlying density dependence, and the asymptotic spatially averaged population density tends to a constant value as the size of the landscape becomes very large. In this phase, the synchronization order parameter vanishes. Only at sufficiently low noise levels can cycling emerge in asymptotic spatially averaged population density. In this phase, the asymptotic system is no longer invariant to odd-period time translations. A discrete twofold symmetry is broken in the transition from incoherence to synchrony, and this is the symmetry breaking pattern of the Ising universality class^22^,^23^,^24^,^25^.

### Asymptotic measures of spatial synchrony

We define new measures of spatial synchrony that complement existing approaches^3^,^5^,^6^,^7^,^8^,^9^,^10^,^11^,^12^,^13^,^14^,^15^,^16^,^27^,^32^,^49^,^50^,^51^,^52^,^53^,^54^,^55^,^56^. Each asymptotic measure is identical to a statistic in the Ising model if the *m*_*j*,*t*_ are the Monte Carlo trajectories of a local spin rather than a time series of local two-cycle amplitudes. Each transient measure corresponds to an asymptotic measure but can be estimated at a single generation of the population model and does not require the existence of a long nor stationary time series.

*Integrated autocorrelation time*. This statistic measures the characteristic timescale over which correlations in trajectories of the instantaneous synchronization decay. Our estimates and error bars, as plotted in Supplementary Fig. 3, are based on p. 433 of Janke^62^.

*Synchronization order parameter (m_L_).* Because critical transitions occur only in the limit of infinite system size, numerical estimates of the synchronization order parameter on finite-sized *L* × *L* landscapes are denoted with a subscript, *m*_*L*_, and are based on a spatial average of the |*m*_*j*,*t*,*L*_| (see, for example, equation (3) of Janke^62^). The estimate of the synchronization order parameter, as plotted in Fig. 1 and elsewhere, is


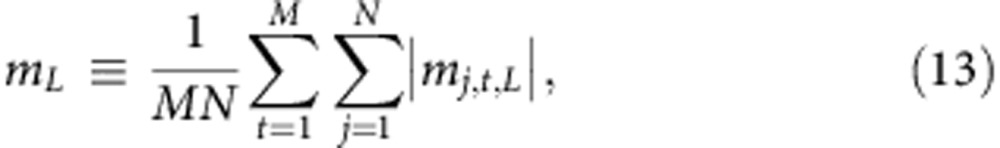


where *N* is the number of samples in the spatial averaging (the number of habitat patches on the landscape) and *M* is the number of samples in the time averaging (the length of the time series). Error bars are estimated based on the integrated autocorrelation time^63^.

*Susceptibility (χ_L_)*. Defined as


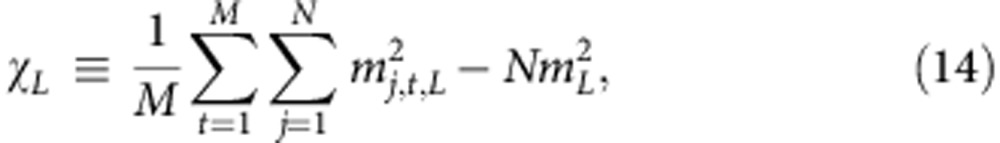


the susceptibility measures the strength of fluctuations in the synchronization order parameter. Error bars are estimated in a block bootstrap^63^ with 100 blocks.

*Fluctuation correlation length (ξ_L_)*. This statistic measures the characteristic length scale over which correlations in local two-cycle amplitudes decay. On a landscape of infinite size, let the displacement between habitat patches *i* and *j* be **r**_*i*,*j*_. The strength of correlations in the *m*_*j*,*t*_, as a function of **r**_*i*,*j*_, is given by the spatially averaged connected correlation function^63^


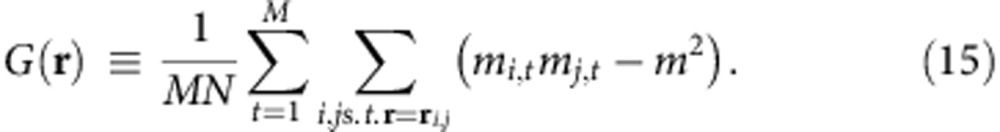


Over large distances, we assume that *G*(**r**) decays exponentially. The fluctuation correlation length is the characteristic length scale of that exponential decay^62^


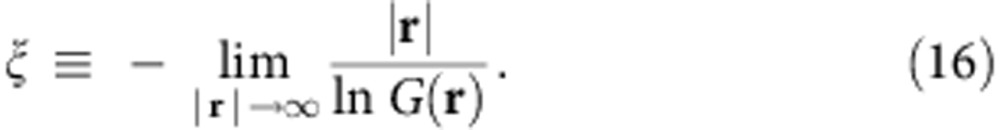


Our estimates of mean values and error bars for the fluctuation correlation length on finite-sized landscapes, *ξ*_*L*_, are based on p. 23 of Janke^64^.

*Critical noise levels (λ_c_)*. Our estimates of critical noise levels, *λ*_*c*_, at the onset of long-range synchronization are based on crossings of Binder's cumulant, *U*^65^. In the 2D Ising model, the critical value, U*, is universal and lies between −1.830 and −1.835 (ref. 39). We estimate U* in each of the four population models and find a value consistent with the Ising result.

### Transient measures of spatial synchrony

Each transient measure of spatial synchrony corresponds to an asymptotic measure but can be estimated at a single generation of the population model and does not require the existence of a long nor stationary time series. Transient measures are defined on a 2D landscape of *N*=*L*^2^ habitat patches. We omit an explicit subscript ‘*L*' on the symbols below to simplify notation.

*Synchronization index (S_t_)*. This statistic is an estimate of the synchronization order parameter windowed in time. We define the index as the absolute value of a two-generation simple moving average of the instantaneous synchronization (equation (10))


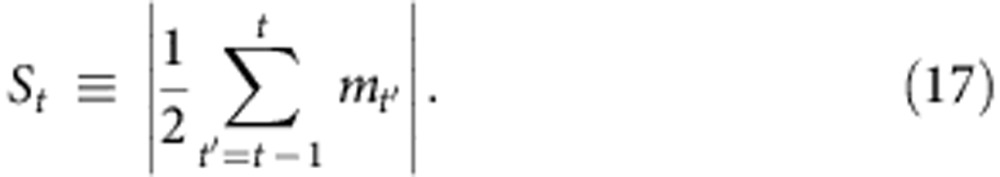


*Susceptibility index (V_t_)*. This statistic is an estimate of susceptibility windowed in both space and time. We define a square spatial window covering *N*′=*L*/2 × *L*/2 habitat patches of the *L* × *L* landscape. With periodic boundary conditions, that window can be placed on the landscape in *N* possible ways. Let the *k*th placement of the spatial window cover *N*′ habitat patches with their locations indexed by the elements of 
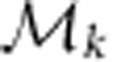
. For each placement in each generation, we calculate the average value of the two-cycle amplitudes (equation (10)) that fall within the spatial window


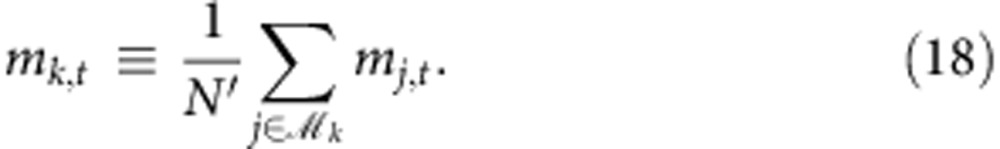


Over two generations, we obtain 2*N* estimates of *m*_*k*,*t*_. The absolute value of the mean of the estimates is simply the synchronization index defined above. The susceptibility index is given by


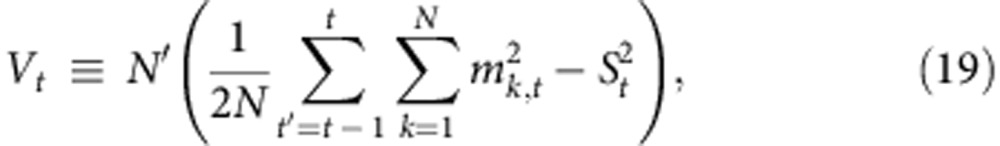


and provides a measure of variability in local synchronization.

*Correlation length index (CL_t_)*. This statistic defines a characteristic length scale for the size of synchronized clusters using the same approach to spatial and temporal windowing as in the definition of the susceptibility index. In the absence of long-range correlations and a long time series, we cannot obtain a good estimate of the long-distance exponential decay of the connected correlation function (equation (15)) that defines the finite-sized fluctuation correlation length, *ξ*_*L*_ (equation (16)). Instead, we define CL_*t*_ to be a length scale determined by the short-distance exponential decay of a spatially windowed correlation function. On the *k*th spatial window covering *N*′=*L*/2 × *L*/2 habitat patches at generation *t* (see the discussion of spatial windowing in the definition of *V*_*t*_ above), we define the correlation function, *G*_*k*,*t*_(**r**), as


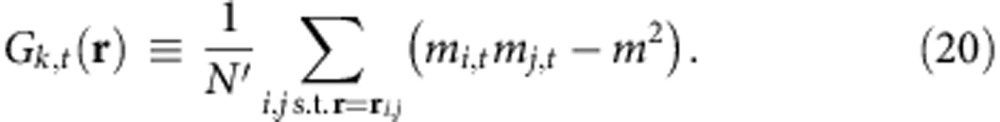


This function can be averaged over *N* possible placements of the spatial window in each of two generations, defining *G*_*t*_(**r**) as


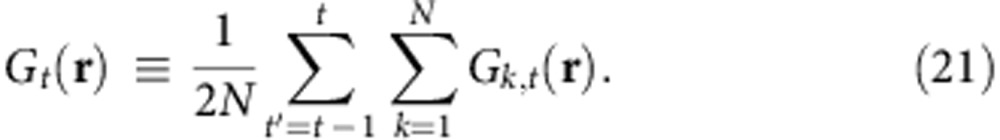


We hypothesize that *G*_*t*_(**r**) decays exponentially even over short distances such that


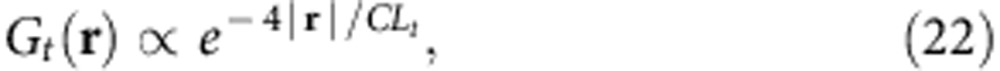


and that CL_*t*_ can be estimated simply as


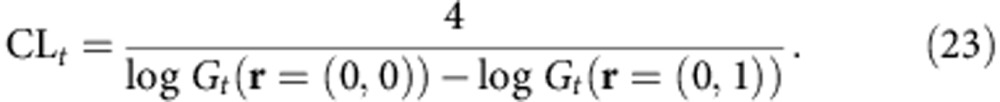


The overall scale factor of 4 is chosen such that CL_*t*_ is roughly equal to the typical diameter of a synchronized cluster. In a sudden transition from incoherence to cluster formation and growth, a typical trajectory of CL_*t*_ lies on the curve CL_*t*_∝*t*^1/2^, as shown in Fig. 4c. This is the expected power-law scaling for coarsening behaviour in the Ising model with non-conserved dynamics^22^,^23^,^24^,^25^.

### Finite-size scaling and evidence of Ising universality

Strictly speaking, the Ising critical transition occurs only on an infinite lattice of spins. Nonetheless, critical behaviour can be studied in numerical Monte Carlo simulations of finite-sized lattices^22^,^23^,^24^,^25^. Finite-size scaling theory has previously been used to provide strong numerical evidence of Ising (or in some cases, ‘Ising-like') universality in the critical transitions of probabilistic cellular automata^36^ and chaotic map lattices^37^,^38^,^39^,^43^. Here, we apply similar methods to study critical transitions in coupled maps of non-chaotic, noisy two-cycles with applications to ecology. We first define the analogue of reduced temperature in our noisy population models and then introduce our finite-size scaling hypothesis.

*Reduced control parameters*. In studies of the Ising model, the reduced temperature (*τ*_*T*_) is defined as (*T*–*T*_c_)/*T*, where *T* is the temperature and *T*_c_ is the critical temperature. To investigate the Ising universality of noise-induced critical transitions in ecological models, we define the reduced noise level (*τ*_*λ*_) as (*λ*–*λ*_c_)/*λ*, where *λ* is the noise level and *λ*_c_ is the critical noise level. If we want to refer to either *τ*_*T*_ or *τ*_*λ*_, we will simply use the symbol *τ* and call this the reduced control parameter.

*Data collapse for ecological statistics*. Near a critical transition in the Ising model and the ecological models, we gather finite-size measurements of the synchronization order parameter, *m*_*L*_, susceptibility, *χ*_*L*_, and fluctuation correlation length, *ξ*_*L*_, for various landscape sizes, *L*, and reduced control parameter values, *τ*. For each model, we find that these measurements can be scaled to lie on a function, *F*_*m*_, *F*_*χ*_ or *F*_*ξ*_, that is independent of both the model and the landscape size^22^,^23^,^24^,^25^


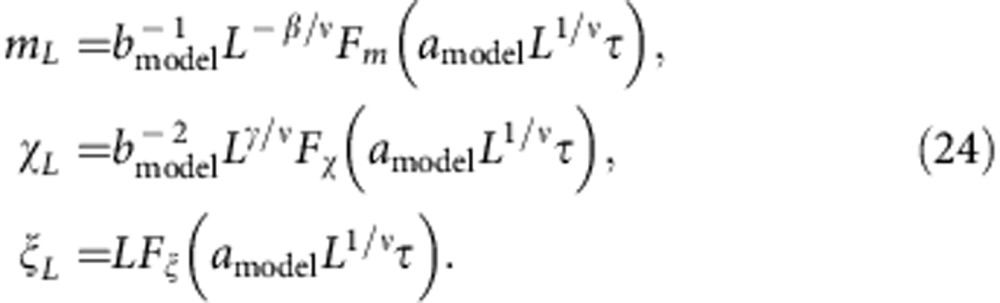


The powers of *L* are determined by the 2D Ising critical exponents (*ν*=1, *β*=1/8, *γ*=7/4) and are model independent. The model-dependent scale factors are *a*_model_ and *b*_model_, where *a*_model_ is a scaling of the reduced noise level and *b*_model_ is a scaling of local two-cycle amplitudes


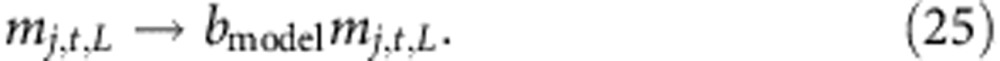


The asymptotic measures of spatial synchrony, *m*_*L*_, *χ*_*L*_ and *ξ*_*L*_, defined above in terms of the *m*_*j*,*t*,*L*_, scale with *b*_model_ as


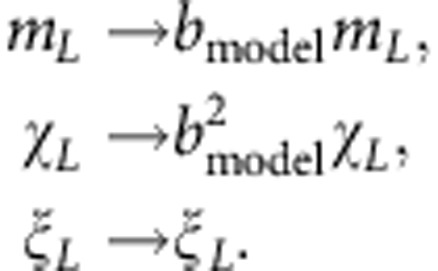


We choose *a*_Ising_=*b*_Ising_=1 without loss of generality. For the ecological models, corrections to scaling and quantitative estimates of critical exponents will be published elsewhere.

### Monte Carlo simulations

Our numerical methods for estimating asymptotic statistics characterizing noisy ecological populations are based on well-established methods in the statistical physics literature on Monte Carlo simulations^62^,^63^,^64^,^65^. In each Monte Carlo simulation, we iterate equation (4) or (7), 10 for a burn-in period of *T*_burnin_ generations before running for *T*_steps_ generations and gathering *M* samples of each statistic at regular intervals. In all simulations, initial population densities are drawn from a normal distribution with mean *μ*_0_ and s.d. *σ*_0_ (negative numbers, if drawn, are thrown out), but results are independent of initial conditions, so long as local population densities are nonzero. Details follow on the Monte Carlo simulations used to estimate the statistics plotted in the main text and Supplementary Information.

Fig. 1: (**a**,**b**) On the basis of Monte Carlo simulations, with *T*_burnin_=2 × 10^6^, *T*_steps_=2 × 10^7^ and *M*=1 × 10^5^, of the Ricker model, with *r*=2.3, 
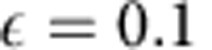
 and *λ* one of 64 evenly spaced values on the interval [0.12,0.18]. Snapshots are shown in **c** for *λ*=0.145. Initial population densities are drawn from a normal distribution with *μ*_0_=0.50 and *σ*_0_=0.1. (**d**,**e**) On the basis of Monte Carlo simulations, with *T*_burnin_=2 × 10^6^, *T*_steps_=2 × 10^7^ and *M*=1 × 10^5^, of the host–parasitoid model, with *r*=1.5, 
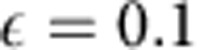
, *a*=20 and *λ* one of 64 evenly spaced values on the interval [0.3,0.5]. Snapshots are shown in **f** for *λ*=0.376. Initial population densities are drawn from a normal distribution with *μ*_0_=0.50 and *σ*_0_=0.1.

Fig. 2: (**a**–**c**) On the basis of Monte Carlo simulations, with *T*_burnin_=2 × 10^7^, *T*_steps_=2 × 10^8^ and *M*=1 × 10^5^, of the Ricker model, with *r*=2.3, 
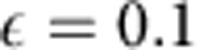
. We scanned 72 evenly spaced values of *λ* on the interval [0.135,0.150] but plotted statistics over a smaller range closer to the critical transition. Initial population densities are drawn from a normal distribution with *μ*_0_=0.50 and *σ*_0_=0.1. The critical noise level, *λ*_*c*_=0.14131(4), is estimated from the critical value of Binder's cumulant and used to calculate the reduced noise level as plotted on the horizontal axis. (**d**–**f**) The points and error bars are the *N*=4,096 series in panels b, d and f of Supplementary Figs 4–8 (see details below), after a model-dependent scaling of the horizontal and vertical axes by *a*_model_ and *b*_model_. Without loss of generality, we define *a*_Ising_=*b*_Ising_=1. Then we estimate *a*_Ricker_=2.60, *a*_host–parasitoid_=3.15, *a*_Logistic_=2.85, *a*_Ricker–Moore_=2.65, and *b*_Ricker_=2.028, *b*_host–parasitoid_=4.382, *b*_Logistic_=7.228, *b*_Ricker–Moore_=2.179.

Fig. 3: The phase boundary plotted in this figure is an interpolation of the maximal values of fluctuation correlation length in Supplementary Fig. 10 (see details below).

Fig. 4: On the basis of Monte Carlo simulations, with *T*_burnin_=0 and *T*_steps_=10, of the Ricker model, with 
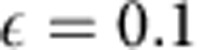
 and *λ*=0.12. For the signal (red lines), *r*=2.3; for the baseline behaviour (blue lines), *r*=1.5. In each scenario, trajectories of the synchronization, susceptibility and correlation length indices are plotted for 1,000 initial population configurations. Initial population densities are drawn from a normal distribution with *μ*_0_=1.00 and *σ*_0_=0.1.

Supplementary Fig. 1: same as Fig. 1a–c but for the Logistic model, with *r*=3.3, *ɛ*=0.1 and *λ* one of 64 evenly spaced values on the interval [0.04,0.07]. Snapshots are shown in panel (C) for *λ*=0.0519.

Supplementary Fig. 2: (A,B) same as Fig. 1 a,b but for the Ricker–Moore model, with *r*=3.3, *ɛ*=0.1 and *λ* one of 64 evenly spaced values on the interval [0.13,0.19]. Snapshots are shown in panel (C) for *λ*=0.154.

Supplementary Fig. 3: integrated autocorrelation times of the synchronization order parameter are estimated from the same Monte Carlo simulations used to generate Fig. 1.

Supplementary Fig. 4: panels D–F are the same as Fig. 2 a–c. Panels A–C in this figure plot the same statistics without finite-size scaling.Supplementary Fig. 5: same as Supplementary Fig. 4 but for the Host–Parasitoid model, with *r*=1.5, *ɛ*=0.1, *α*=20 and *λ* one of 72 evenly spaced values on [0.347,0.397]. Initial population densities, for both host and parasitoid, are drawn from a normal distribution with 
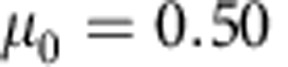
 and 
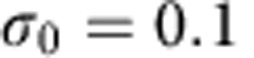
. The critical noise level is 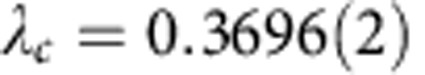.

Supplementary Fig. 6: same as Supplementary Fig. 4 but for the Logistic model, with with 
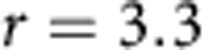
, 
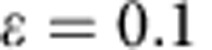
 and 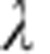 one of 72 evenly spaced values 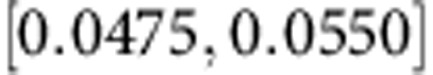. The critical noise level is 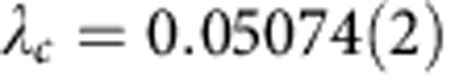.

Supplementary Fig. 7: same as Supplementary Fig. 4 but for the Ricker–Moore model, with 
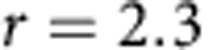
, 
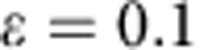
 and 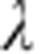 one of 72 evenly spaced values on 
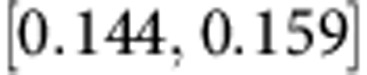
. The critical noise level is 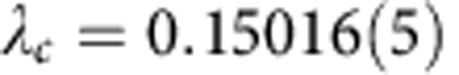.

Supplementary Fig. 8: based on simulations of the 2D Ising model using the Wolff algorithm[63], with 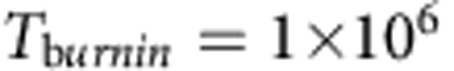, 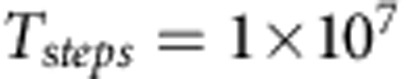 and 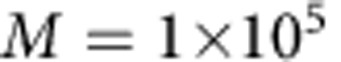, and 
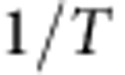
 (in units where Botzmann's constant is set to unity) one of 64 evenly spaced values on the interval 
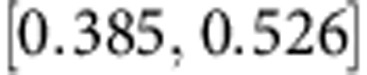
. Initial spin configurations are fully aligned (all spin values are 
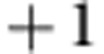
). The critical temperature and theoretical predictions for an infinite lattice are well known in the physics literature^22^,^23^,^24^,^25^.

Supplementary Fig. 9: same as Fig. 2 d–f but for the A–C 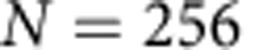 and D–F 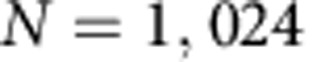 series.

Supplementary Fig. 10: based on Monte Carlo simulations, with 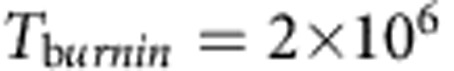, 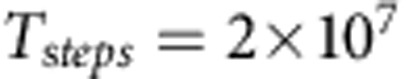 and 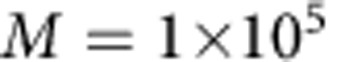, of the Ricker model where 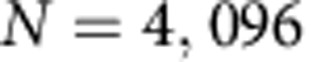 and 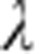 is one of 26 evenly spaced values on the interval 
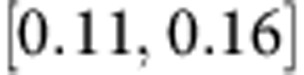
. (A) 
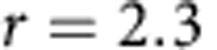
 and 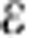 is one of 30 evenly spaced values on 
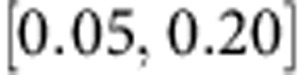
. (B) 
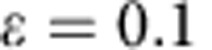
 and 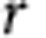 is one of 30 evenly spaced values on 
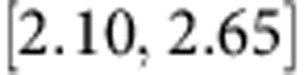
.

## Author contributions

This study has its origins in the lab of A.H. The results are the product of a tight-knit interdisciplinary collaboration among the three authors. J.M. and A.H. contributed to the development and analysis of the results, and to the writing and editing of the manuscript. A.E.N. developed analytical results, ran numerical simulations and took primary responsibility for the manuscript.

## Additional information

**How to cite this article:** Noble, A. E. *et al.* Emergent long-range synchronization of oscillating ecological populations without external forcing described by Ising universality. *Nat. Commun.* 6:6664 doi: 10.1038/ncomms7664 (2015).

## Supplementary Material

Supplementary Information Supplementary Figures 1-10.
