# Supplementary material for: Emergent long-range synchronization of oscillating ecological populations without external forcing described by Ising universality
Source: Nat Commun. 2015 Apr 8;6:6664. doi: 10.1038/ncomms7664 (PMC4403441; doi:10.1038/ncomms7664)
Supplement: Supplementary Information — Supplementary Figures 1-10. [file ncomms7664-s1.pdf]

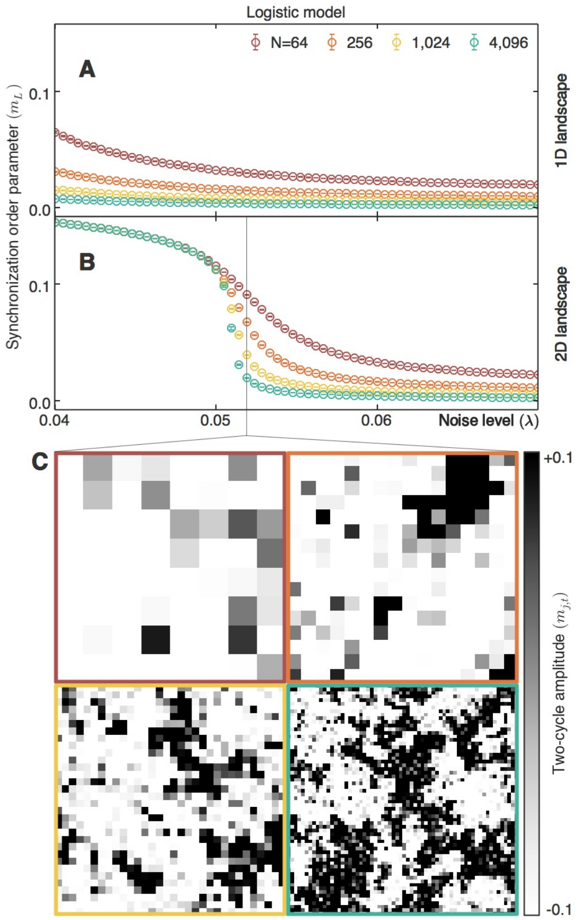

Supplementary Figure 1 | Critical transition at the onset of collective synchronization in the spatial Logistic model on 2D landscapes. Long-range synchronization is expected to emerge independent of the details of the quadratic map regulating local density dependence. (A) On large 1D landscapes (large $N=L$) with short-range dispersal and nonzero uncorrelated environmental noise levels (λ), collective synchronization of spatial populations cannot emerge from local dispersal alone. (B) On 2D landscapes (where $N=L^2$), collective synchronization can emerge from local dispersal at a noise-induced transition. A continuous change in numerical estimates of the synchronization order parameter, m_L, from near zero (the disordered, incoherent phase) to larger values (the ordered, synchronous phase), sharpens as the size of the landscape increases. (C) Near the transition, on 2D landscapes ranging in size from $N=64$ (upper-left) to 4,096 (lower-right), density plots of local two-cycle amplitudes, the $m_{j,t}$, show emergent long-range order in the fractal coexistence of synchronized habitat patches. In statistical physics, a continuous transition from a disordered phase to a phase ordered by emergent long-range correlations defines a “critical transition”. Critical transitions at the onset of collective synchronization also occur in the 2D Ricker, Host-Parasitoid, and Ricker-Moore models. Note that symbols are displayed with s.e.m. error bars but actual error bars are often much smaller than the symbol size.

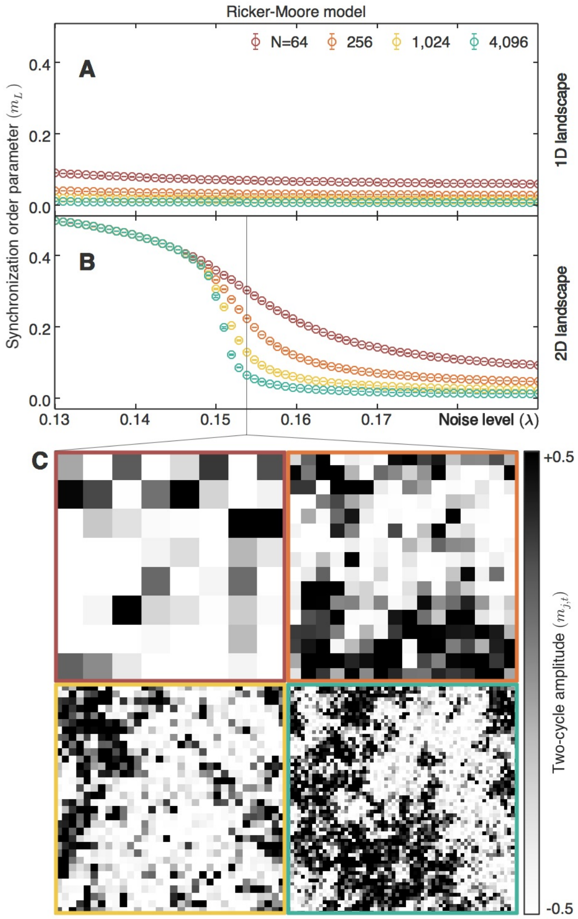

Supplementary Figure 2 | Critical transition at the onset of collective synchronization in the spatial Ricker-Moore model on 2D landscapes. Long-range synchronization is expected to emerge independent of the details of the local dispersal dynamics. (A) On large 1D landscapes (large $N{=}L$) with short-range dispersal and nonzero uncorrelated environmental noise levels (λ), collective synchronization of spatial populations cannot emerge from local dispersal alone. (B) On 2D landscapes (where $N{=}L^2$), collective synchronization can emerge from local dispersal at a noise-induced transition. A continuous change in numerical estimates of the synchronization order parameter, m_L, from near zero (the disordered, incoherent phase) to larger values (the ordered, synchronous phase), sharpens as the size of the landscape increases. (C) Near the transition, on 2D landscapes ranging in size from $N{=}64$ (upper-left) to 4,096 (lower-right), density plots of local two-cycle amplitudes, the $m_{j,t}$, show emergent long-range order in the fractal coexistence of synchronized habitat patches. In statistical physics, a continuous transition from a disordered phase to a phase ordered by emergent long-range correlations defines a “critical transition”. Critical transitions at the onset of collective synchronization also occur in the 2D Ricker, Host-Parasitoid, and Logistic models. Note that symbols are displayed with s.e.m. error bars but actual error bars are often much smaller than the symbol size.

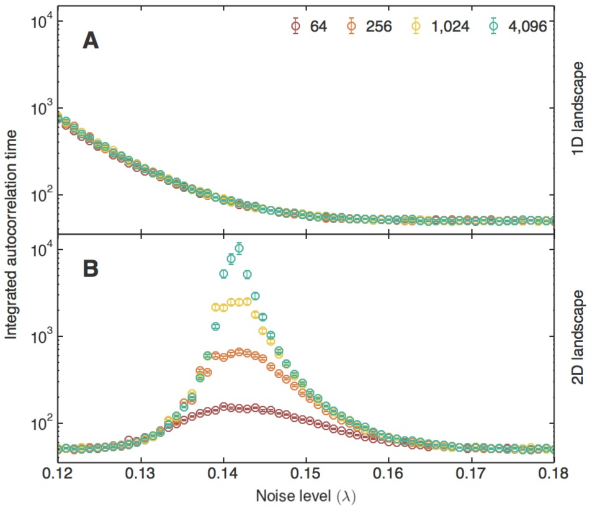

Supplementary Figure 3 | Critical slowing down in the Ricker model. The integrated autocorrelation times, plotted here as a function of noise level (λ), correspond to estimates of the synchronization order parameter in Fig. 1. (A) On 1D landscapes, integrated autocorrelation time increases monotonically with decreasing noise level and does not exhibit a strong dependence on the number of habitat patches (N). (B) On 2D landscapes, the integrated autocorrelation time exhibits a spike near the critical transition in the synchronization order parameter, signaling critical slowing down in the dynamics of spatial synchrony. The spike becomes more pronounced as the number of habitat patches increases, in agreement with the behavior of the dynamical Ising model. Note that symbols are displayed with s.e.m. error bars but actual error bars are often much smaller than the symbol size.

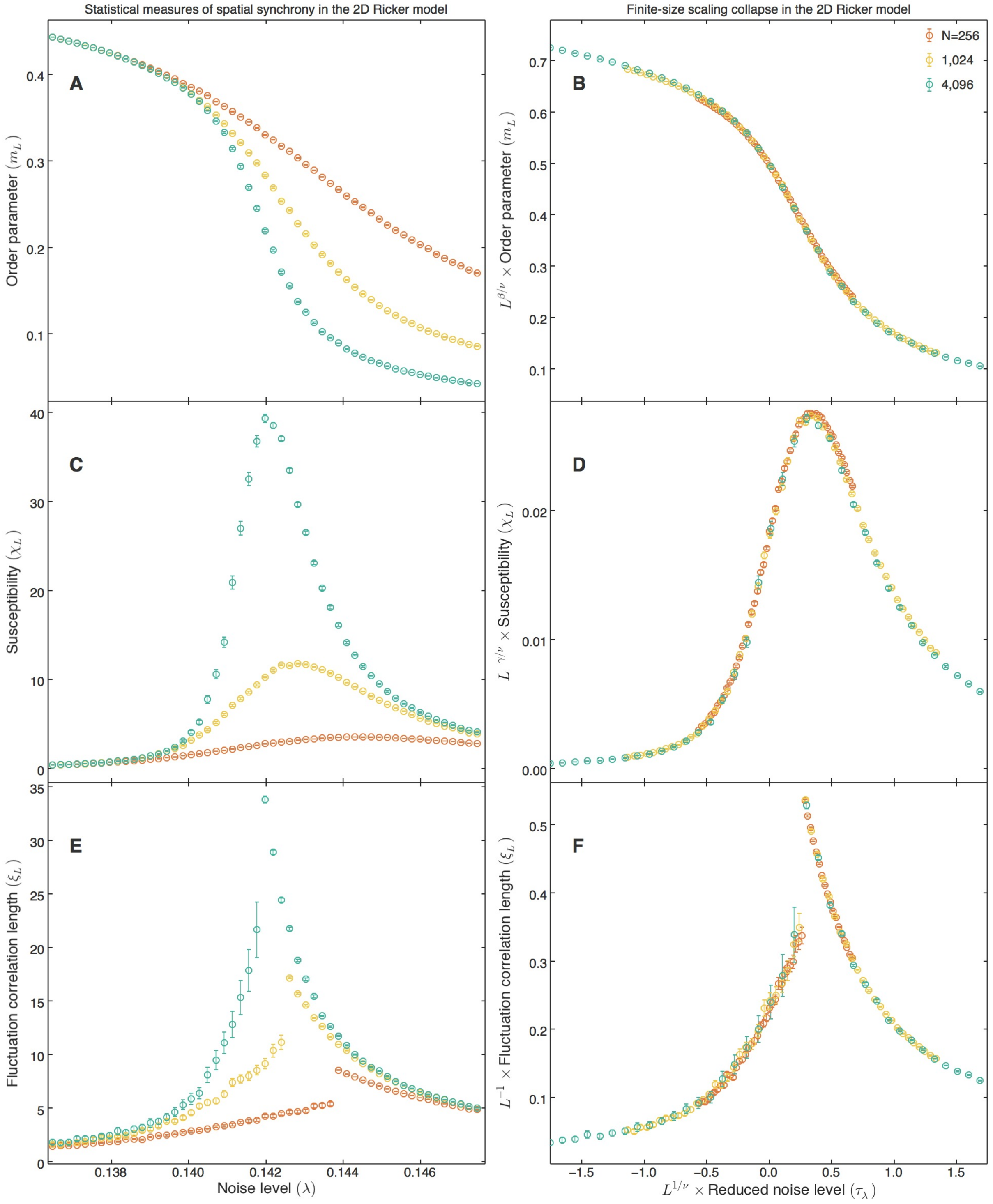

Supplementary Figure 4 | Evidence of Ising universality in the 2D Ricker model. Each unscaled measurement, plotted as a function of noise level λ in the left-hand column of plots, corresponds to a point along the finite-size scaling curves, plotted as a function of reduced noise level τ_λ in the right-hand column. (Results in the right-hand column are also shown in Fig. 2A-C). When critical exponents in the 2D Ising universality class (ν=1, β=1/8, γ=7/4) are assumed, estimates of the (A-B) synchronization order parameter (m_L), (C-D) susceptibility (χ_L), and (E-F) fluctuation correlation length (ξ_L), for three different landscape sizes, collapse onto universal curves (curves independent of landscape size, N=L^2) near the onset of collective synchronization. The same results can be found in all the ecological models that we analyze. These results provide strong evidence that the transition from incoherence to collective synchronization is a critical transition in the Ising universality class. Note that symbols are displayed with s.e.m. error bars but actual error bars are often much smaller than the symbol size.

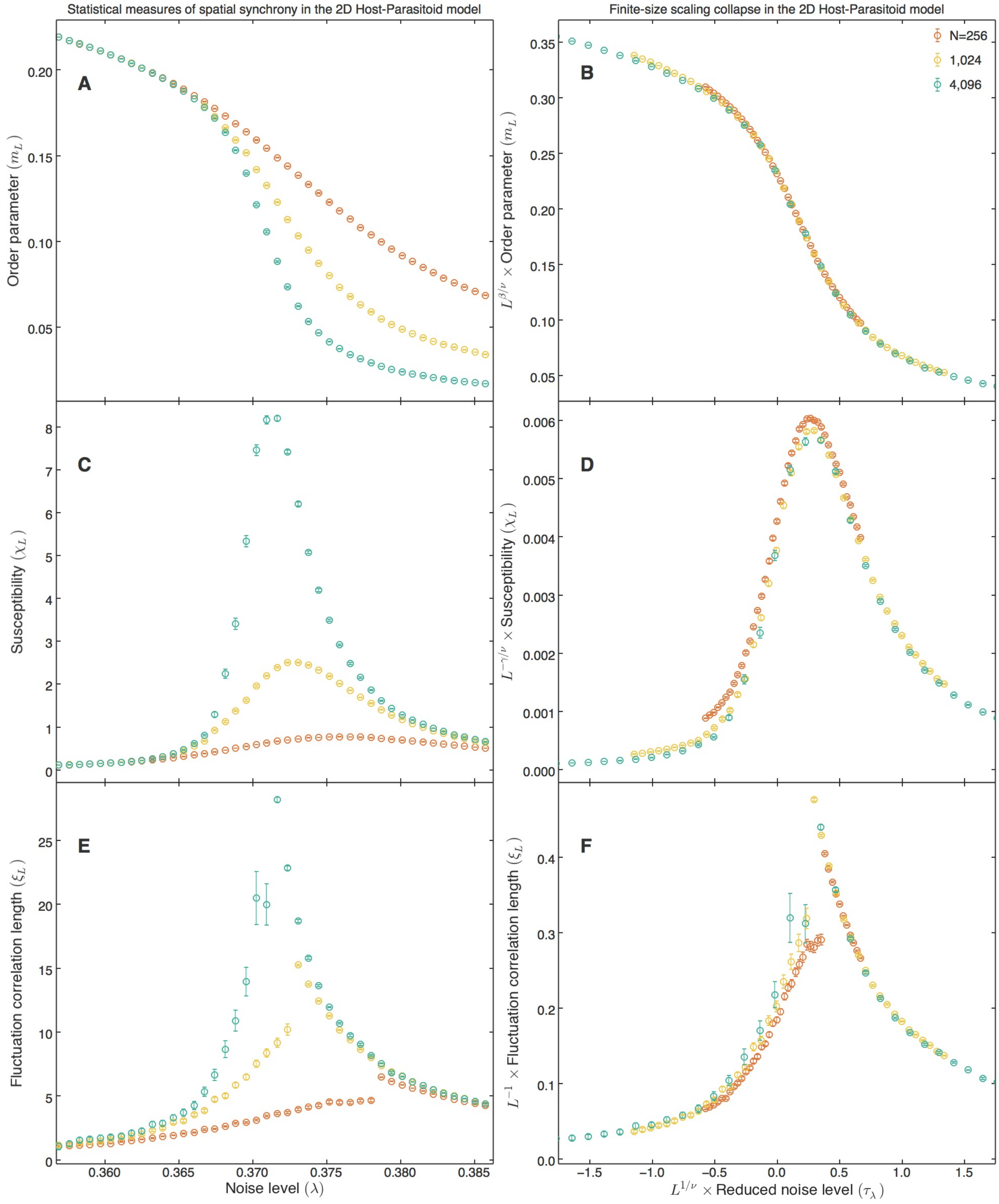

Supplementary Figure 5 | Evidence of Ising universality in the 2D Host-Parasitoid model. Ising universality at the onset of collective synchronization can be found in mechanistic models with more than one species. Each unscaled measurement, plotted as a function of noise level λ in the left-hand column of plots, corresponds to a point along the finite-size scaling curves, plotted as a function of reduced noise level τ_λ in the right-hand column. When critical exponents in the 2D Ising universality class (ν=1, β=1/8, γ=7/4) are assumed, estimates of the (A-B) synchronization order parameter (m_L), (C-D) susceptibility (χ_L), and (E-F) fluctuation correlation length (ξ_L), for three different landscape sizes, collapse onto universal curves (curves independent of landscape size, N=L^2) near the onset of collective synchronization. The same results can be found in all the ecological models that we analyze. These results provide strong evidence that the transition from incoherence to collective synchronization is a critical transition in the Ising universality class. Note that symbols are displayed with s.e.m. error bars but actual error bars are often much smaller than the symbol size.

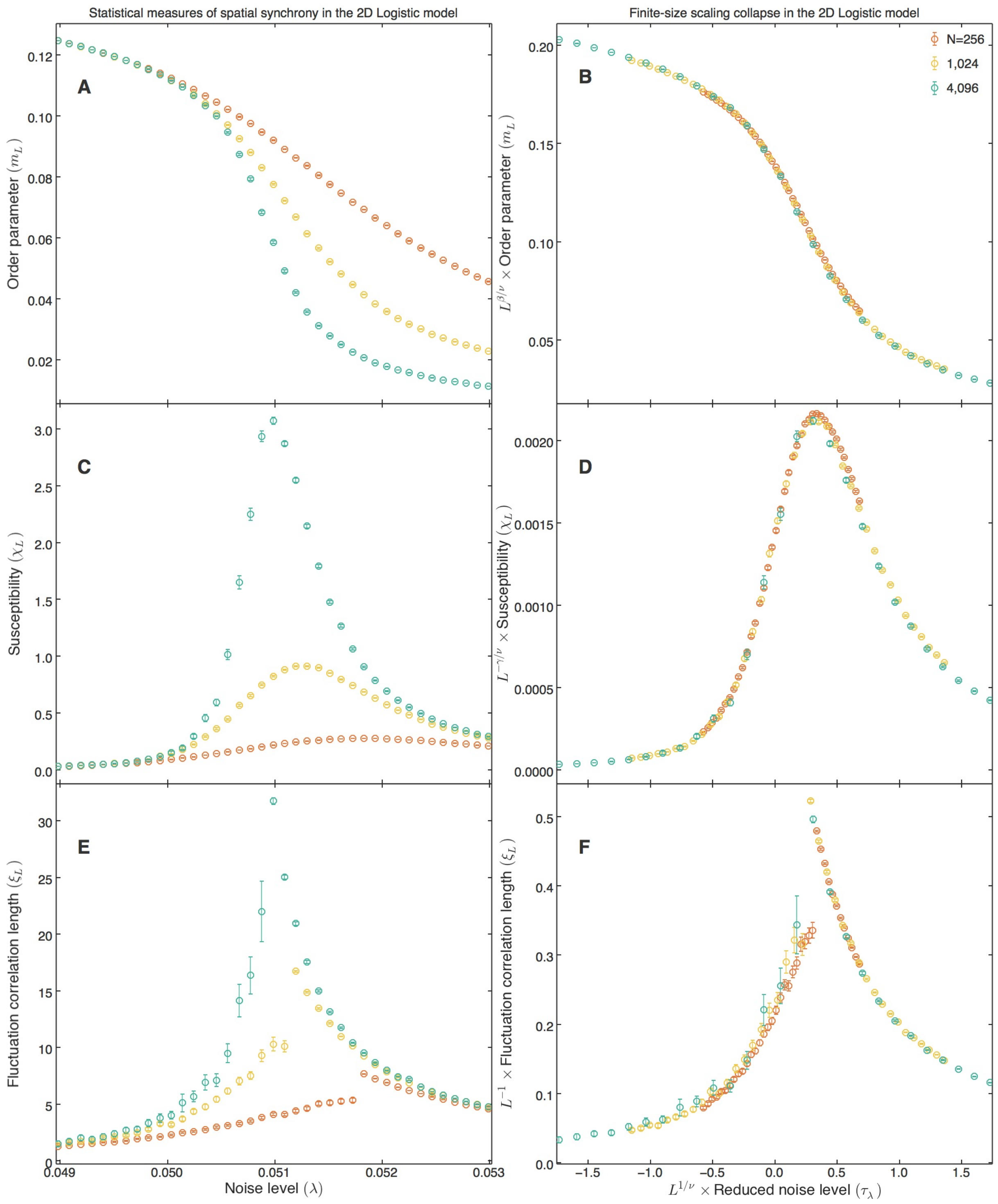

Supplementary Figure 6 | Evidence of Ising universality in the 2D Logistic model. Ising universality at the onset of collective synchronization is robust to variations in the quadratic map regulating local density dependence. Each unscaled measurement, plotted as a function of noise level λ in the left-hand column of plots, corresponds to a point along the finite-size scaling curves, plotted as a function of reduced noise level τ_λ in the right-hand column. When critical exponents in the 2D Ising universality class (ν=1, β=1/8, γ=7/4) are assumed, estimates of the (A-B) synchronization order parameter (m_L), (C-D) susceptibility (χ_L), and (E-F) fluctuation correlation length (ξ_L), for three different landscape sizes, collapse onto universal curves (curves independent of landscape size, $N{=}L^2$) near the onset of collective synchronization. The same results can be found in all the ecological models that we analyze. These results provide strong evidence that the transition from incoherence to collective synchronization is a critical transition in the Ising universality class. Note that symbols are displayed with s.e.m. error bars but actual error bars are often much smaller than the symbol size.

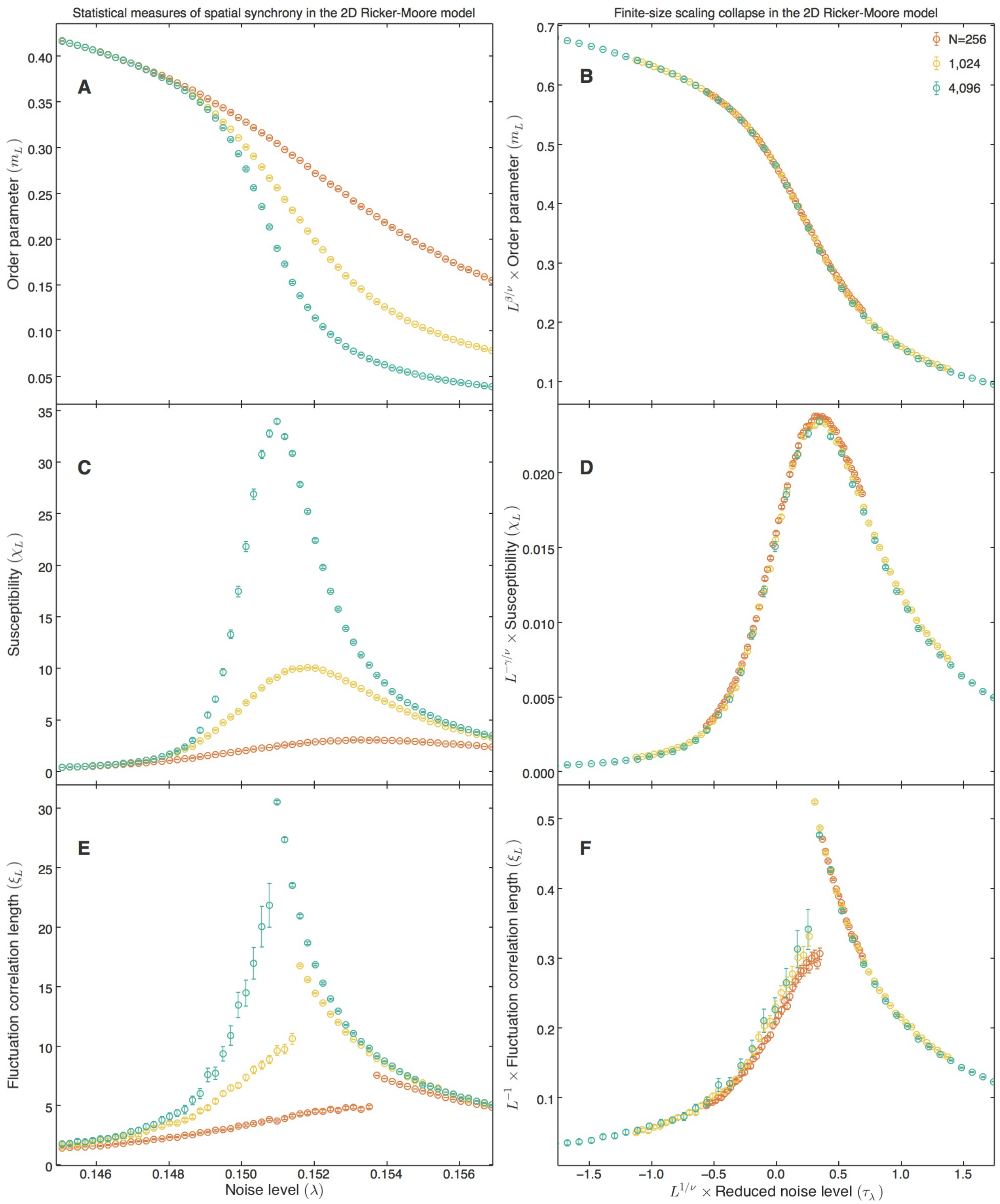

Supplementary Figure 7 | Evidence of Ising universality in the 2D Ricker-Moore model. Ising universality at the onset of collective synchronization is robust to variations in the local dispersal kernel. Each unscaled measurement, plotted as a function of noise level λ in the left-hand column of plots, corresponds to a point along the finite-size scaling curves, plotted as a function of reduced noise level τ_λ in the right-hand column. When critical exponents in the 2D Ising universality class (ν=1, β=1/8, γ=7/4) are assumed, estimates of the (A-B) synchronization order parameter (m_L), (C-D) susceptibility (χ_L), and (E-F) fluctuation correlation length (ξ_L), for three different landscape sizes, collapse onto universal curves (curves independent of landscape size, N=L^2) near the onset of collective synchronization. The same results can be found in all the ecological models that we analyze. These results provide strong evidence that the transition from incoherence to collective synchronization is a critical transition in the Ising universality class. Note that symbols are displayed with s.e.m. error bars but actual error bars are often much smaller than the symbol size.

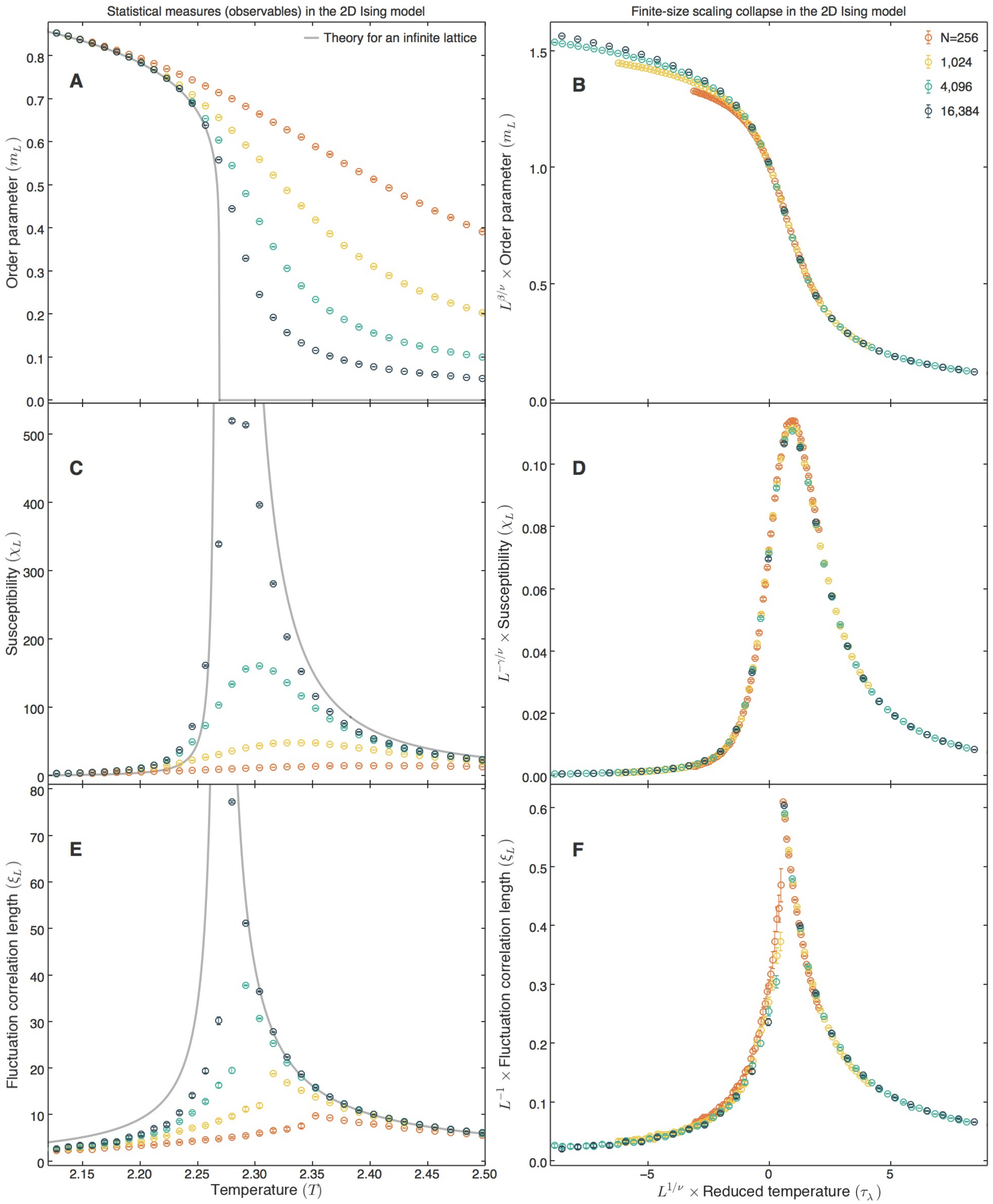

Supplementary Figure 8 | Finite-size scaling near the critical point of the 2D Ising model. Each unscaled measurement, plotted as a function of temperature T in the left-hand column of plots, corresponds to a point along the finite-size scaling curves, plotted as a function of reduced noise level τ_T in the right-hand column. Theoretical predictions on an infinite lattice are plotted for comparison in the left-hand column. When critical exponents in the 2D Ising universality class (ν=1, β=1/8, γ=7/4) are assumed, estimates of the (A-B) order parameter (m_L), (C-D) susceptibility (χ_L), and (E-F) fluctuation correlation length (ξ_L), for four different lattice sizes, collapse onto universal curves (curves independent of lattice size, N=L^2) near the onset of long-range order. The N=4,096 estimates are plotted in Fig. 2D-F. Note that symbols are displayed with s.e.m. error bars but actual error bars are often much smaller than the symbol size.

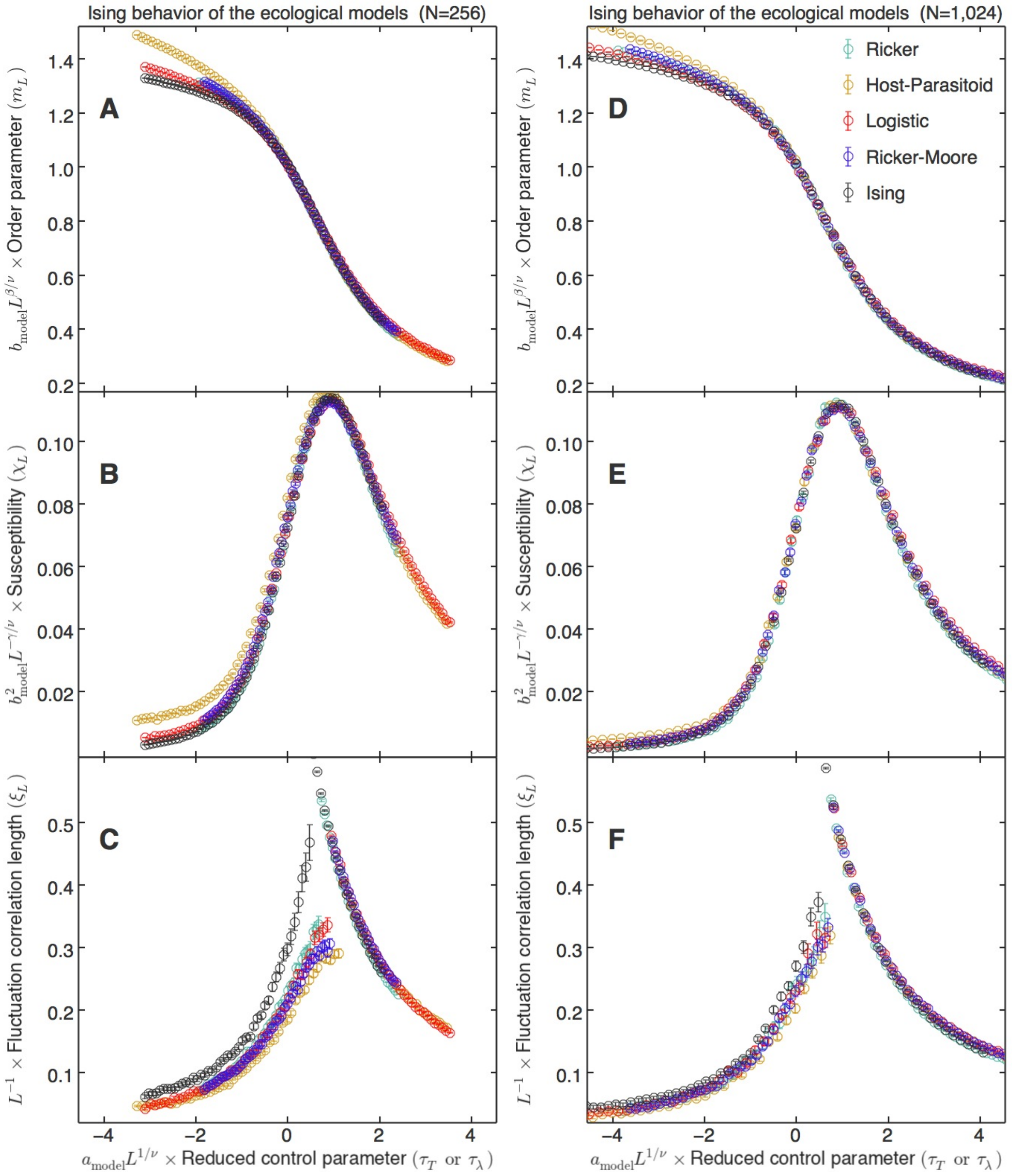

Supplementary Figure 9 | Ising universality of the ecological models on 2D landscapes near the onset of collective synchronization. Scaled measurements of the order parameter (m_L), susceptibility (χ_L), and fluctuation correlation length (ξ_L) for diverse ecological models and 2D landscape sizes (where $N{=}L^2$) coincide with Ising model results, showing the existence of universal behavior. The scaled collapse of ecological results onto the corresponding Ising results is shown for (A-C) $N{=}256$ and (D-F) $N{=}1{,}024$. These plots demonstrate that, on landscape sizes of ecological interest, the Ising statistics remain a good approximation to the corresponding population statistics. The scaling parameters, a_{model} and b_{model}, are chosen separately for each model; without loss of generality, $a_{\mathrm{Ising}}{=}b_{\mathrm{Ising}}{=}1$. The Ising "critical exponents" are $\nu{=}1$, $\beta{=}1/8$, $\gamma{=}7/4$. These results present strong evidence that the onset of collective synchronization is a critical transition in the Ising universality class. Universality explains the robustness and broad applicability of our results: long-range synchronization and power-law scalings emerge from local dispersal independent of the details of local dynamics, including population regulation, dispersal, and landscape connectivity. Note that symbols are displayed with s.e.m. error bars but actual error bars are often much smaller than the symbol size.

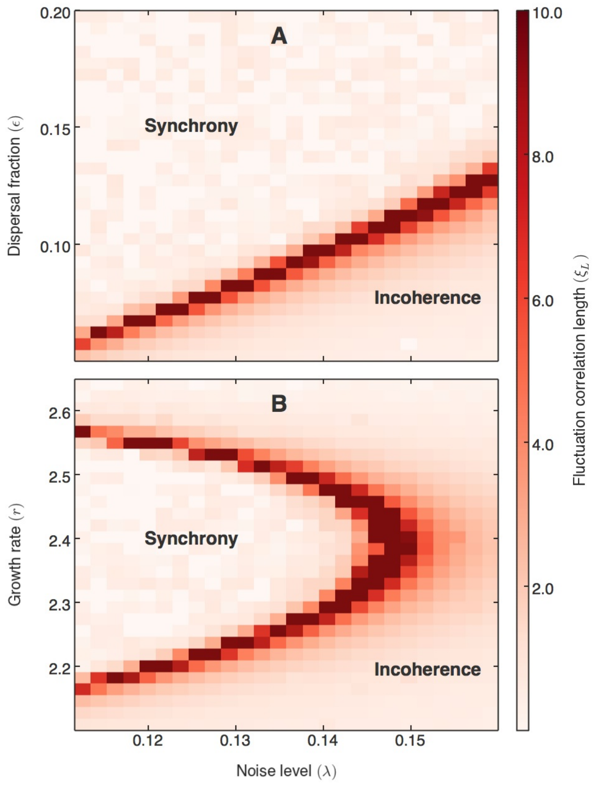

Supplementary Figure 10 | Estimating cross-sections of the critical boundary separating phases of incoherence and collective synchronization in the 2D Ricker model. The parameters of the Ricker model are dispersal fraction (ϵ), growth rate (r), and noise level (λ). For any given choice of parameters, there is a single asymptotic phase: either incoherence (with synchronization order parameter zero) or synchrony (with synchronization order parameter nonzero). These two phases are separated by a boundary of Ising critical transitions. (A) A density plot of fluctuation correlation length estimates based on Monte Carlo simulations on 2D landscapes of N=L^2=4,096 habitat patches. The growth rate is fixed at 2.3. (B) A density plot of fluctuation correlation length estimates for a fixed dispersal fraction of ϵ=0.1 and N=4,096 habitat patches. The critical phase boundary can be approximated by interpolating among points in the parameter space where the fluctuation correlation length is maximal.
